# Computational Landscape in Drug Discovery: From AI/ML Models to Translational Application

**DOI:** 10.1155/sci5/1688637

**Published:** 2025-11-24

**Authors:** Deepak Sharma, Madhu Anabala, V. Vanitha Jain, Mukul Shyam, Sabina Evan Prince, Rajiniraja Muniyan

**Affiliations:** School of Bio-Sciences and Technology, Vellore Institute of Technology, Vellore 632014, Tamil Nadu, India

**Keywords:** artificial intelligence, computational modeling, deep learning, drug discovery, machine learning

## Abstract

The combination of artificial intelligence (AI) and machine learning (ML) in drug discovery has significantly transformed traditional pharmaceutical research by enabling data-driven decision-making, accelerating the identification of hits, and improving the efficiency of lead optimization. This review provides a comprehensive overview of AI/ML models, including supervised, unsupervised, semisupervised, deep learning, and reinforcement learning approaches and their applications across various stages of drug development, from target identification and virtual screening to de novo molecule design and ADME/T prediction. We highlight widely used ML algorithms, performance evaluation metrics, and AI-driven tools that have become instrumental in modern drug discovery pipelines. Despite rapid advancements, challenges such as limited data availability, heterogeneity, bias, lack of model interpretability, reproducibility concerns, clinical translational barriers, and regulatory uncertainties continue to hinder full-scale adoption. The review also discusses emerging trends, including explainable AI, federated learning, and integration with high-throughput experimental platforms, which offer promising directions for overcoming current limitations. Emphasis is placed on the importance of interdisciplinary collaboration to bridge computational predictions with experimental validation, ensuring robust, ethical, and clinically translatable AI applications in drug development.

## 1. Introduction

The discovery of new drugs is inherently complex, time-consuming, and costly. Developing a new medicine typically requires an average of 10–15 years and an investment exceeding $2.6 billion. Attrition rates are significant at all stages, particularly during clinical trials [1]. High-throughput screening, chemical synthesis, and biological tests are essential components of traditional methodologies; however, their efficacy diminishes when scaling, achieving accuracy, and ensuring reproducibility. The vast quantities of data generated from genomics, proteomics, cheminformatics, and clinical research present both challenges and opportunities for the pharmaceutical industry. In this context, artificial intelligence (AI), particularly machine learning (ML), has significantly transformed the methodologies employed in the discovery of novel drugs [2, 3]. AI encompasses the creation of computer systems that can carry out tasks usually requiring human cognitive abilities, including pattern recognition, reasoning, learning, and making decisions. ML, which falls under the umbrella of AI, allows computers to learn from data and to make decisions or predictions on their own [4].

ML methodologies can be applied in pharmaceutical development to forecast chemical properties, identify drug-target interactions (DTIs), repurpose existing drugs, enhance lead compounds, and generate novel molecules (de novo). These qualities are essential for reducing the effort required in experiments and improving the hit-to-lead ratio. Numerous ML algorithms have been employed in this domain. Support vector machines (SVMs), decision trees (DTs), and random forests (RFs) are examples of supervised learning models [5, 6]. Other approaches consist of unsupervised methods like clustering and dimensionality reduction. Convolutional neural networks (CNNs), recurrent neural networks (RNNs), and graph neural networks (GNNs) are examples of advanced deep learning (DL) models. The use of reinforcement learning (RL) and generative adversarial networks (GANs) in designing and improving new molecules is growing. Tools for natural language processing (NLP), especially transformer-based models like Bidirectional Encoder Representations from Transformers (BERT) and Generative Pre-Training Transformer (GPT), have proven effective in mining biomedical literature, analyzing representations of chemical structures (e.g., SMILES), and producing predictive embeddings [7, 8].

Despite these advancements, several issues remain to be addressed before AI can be effectively utilized in the field of drug discovery. Data availability and quality present ongoing challenges; numerous biomedical datasets are characterized by noise, sparsity, imbalance, or inadequate annotations. These issues complicate model training and diminish its applicability in various contexts [9]. Understanding complex models, particularly DL architectures, remains a significant challenge. This presents a challenge for critical tasks such as safety profiling and clinical decision-making [10]. Ethical and legal concerns arise regarding data privacy, algorithmic fairness, and accountability, particularly when models predict outcomes for individual patients or are utilized in clinical trials. Reproducibility is crucial, as numerous AI-based studies fail to provide public access to their data, code, or standardized evaluation metrics [11]. The prospects for AI in drug development appear promising. Recent advancements such as foundation models, federated learning (FL), multimodal data fusion, and explainable AI (XAI) are anticipated to enhance the reliability, generalizability, and honesty of models. The convergence of AI, quantum computing, automated synthesis platforms, and systems pharmacology may soon enable a fully digital drug development process. Achieving this objective requires collaboration among computational scientists, biologists, chemists, medical professionals, and regulatory bodies to ensure that advancements in AI are transitioned safely and effectively from development to clinical application. This review seeks to provide a thorough overview of the role of AI and ML in drug development in light of these advancements, emphasizing various models and algorithms, key performance metrics, commonly used tools, existing challenges, and future prospects in the field.

## 2. Types of ML Models in Drug Discovery

**Figure 1:**
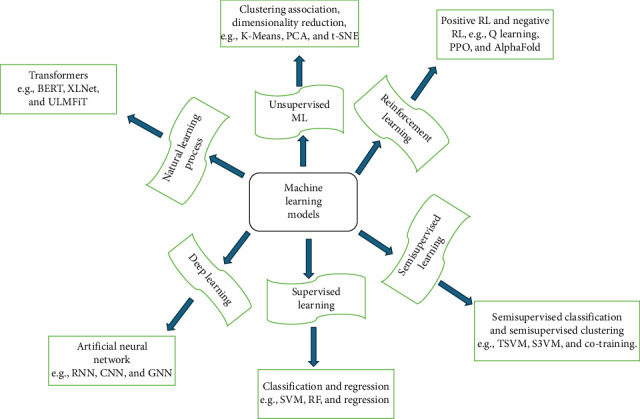
Diagrammatic depiction of several ML models, techniques, and subclasses.

Numerous ML models are applicable in drug discovery. Supervised learning, unsupervised learning, semisupervised learning, RL, DL, and NLP, specifically transformers, are examples of frequently used ML techniques. The following section discusses these ML models. A self-explanatory depiction of these ML models, algorithms, and their subclasses is presented in Figure 1.

### 2.1. Supervised ML (SML)

In a SML structure, models are built on labeled datasets where input features (such as molecular descriptors, fingerprints, or physicochemical properties) are associated with known outputs (such as IC50 values, activity classes, or toxicity). The objective is to create a mapping function that will precisely forecast the outcomes of novel, unidentified data [12]. SML can be classified into two categories: classification and regression [13]. Classification utilizes an algorithm to effectively categorize and differentiate data into several groups, such as determining if a substance is active or inactive. Conversely, regression uses algorithms to forecast a continuous numerical value, such as the binding affinity or inhibition constant of a chemical. The most often employed SML algorithms include “RF, DTs, SVM, Logistic Regression, Naive Bayesian classifier, and Linear Regression,” among others [14]. Numerous QSAR-based investigations utilize SML for the virtual screening of extensive libraries.

### 2.2. Unsupervised ML (USML)

A method known as USML finds and examines patterns in data without knowing the results beforehand. It is used when there is no label on the data [13], concentrating on uncovering latent features or clusters within the data. The basic objective of unsupervised learning is to achieve a more profound understanding of the data by analyzing its distribution [14]. Clustering, association, and dimensionality reduction are classifications within the USML categories [15]. The data are being clustered according to specific patterns, utilizing a clustering technique that includes categories such as “K-Means Clustering, Hierarchical Clustering, and Density-Based Spatial Clustering of Applications with Noise (DBSCAN)” [14]. Dimensionality reduction is a fundamental technique utilized in ML to decrease the number of variables in a dataset while retaining maximal pertinent information. Diverse methodologies are employed in dimensionality reduction, including “Principal Component Analysis, Linear Discriminant Analysis, t-SNE, and Autoencoders” [16].

### 2.3. Semisupervised ML (SSML)

SSML integrates both supervised and USML methods, utilizing partially labeled data [17]. It is utilized when labeled data are insufficient, and annotating additional data would incur significant costs. It entails guiding the model to identify concealed patterns within a limited set of labeled data to facilitate predictions on unlabeled data. Consequently, both labeled and unlabeled data can be utilized to instruct the algorithm, thereby enhancing its performance [13]. A certain number of labeled patterns are used as training data, and the remaining patterns are used as test data when SSML creates a model. Semisupervised classification and semisupervised clustering are the two subtypes of SSML. Because semisupervised classification uses less training data to categorize large amounts of test data, it lessens the need for training data. In semisupervised clustering, we utilize both labeled and unlabeled data alongside pairwise constraints (must-link and cannot-link) to categorize data patterns [17]. Commonly employed methodologies encompass co-training, self-training, and multiview learning [13], as well as transductive support vector machines and semisupervised support vector machines [17].

### 2.4. RL

RL is a method of training a model using a reward and punishment framework, wherein the algorithm acquires behaviors that optimize rewards over time [13]. In the absence of training data, the model learns from its experiences [14]. Deep RL is a technique for synthesizing chemical compounds with specific physical, chemical, and bioactivity characteristics from the ground up. RL is a domain of AI that addresses dynamic decision-making challenges [18]. RL encompasses two categories: positive RL and negative RL. Positive reinforcement occurs when a certain behavior results in greater frequency, intensity, improved performance, and sustained change over an extended duration. Conversely, negative RL involves the enhancement of behavior with the elimination of an adverse condition. Negative RL enhances model behavior [14]. Commonly cited instances of RL encompass Q Learning, Proximal Policy Optimization, Reinforce, and AlphaFold, particularly within the realms of drug discovery and biological methodologies [19].

### 2.5. DL

DL is a type of ML method that learns data representations by using artificial neural networks (ANNs) with several layers of nonlinear processing units [2]. A DL model typically consists of input, output, and hidden layers. Data are entered into the input layer and passes through intermediate hidden levels before reaching the output layer [14]. The most common DL algorithms are “CNN or ConvNet, RNN, Transformers, and GNNs.” Since DL is a key player in the drug discovery and biology domain, DL subclasses, along with their uses, have been discussed in subsequent sections.

### 2.6. Natural Language Processing: Transformers

Transformer-based models are becoming recognized in bioinformatics for addressing sequence-related difficulties, mostly because of the structural parallels between human language and biological sequences. These algorithms utilize extensive unannotated datasets from sources like books and online content via self-supervised learning, wherein they forecast either the subsequent word or a concealed word within a sentence. This method differs from SL, which depends on human-labeled data, thus obviating the necessity for manual annotations. Transformer models' capacity to handle sequences concurrently and capture long-range dependencies enables them to attain superior performance in tasks such as machine translation and question answering [20]. The swift progress in NLP is due to robust DL models like ULMFiT, BERT, XLNet, and several BERT extensions [21].

## 3. Frequent Algorithms Used in ML for Drug Discovery

### 3.1. SVM

SVM is a method for SML that facilitates the classification of compounds, their ranking, and the prediction of property values using regression analysis. SVMs are predominantly employed to predict binary characteristics, like water solubility, synthetic accessibility, or specific activity, as well as to distinguish between medications and nondrugs [22]. The technique makes use of the idea of a hyperplane, which acts as a decision boundary and divides data points into discrete groups. Finding the hyperplane that maximizes the margin is the aim of SVM. SVM can handle data that are not consistently linearly separable and achieve strong generalization performance because of this margin maximization technique [13]. SVM classifiers typically utilize kernel functions like “linear, polynomial, radial basis function (RBF), and sigmoid.” Nonetheless, when the dataset includes extraneous noise, such as overlapping target classes, SVM exhibits suboptimal performance [19].

### 3.2. DT

A DT consists of nodes and the branches that interconnect them in a hierarchical structure. At each node of the tree, a decision is rendered based on certain criteria derived from data analysis [23]. Decisions, their possible results, and the final decision-making process are represented by the nodes, branches, and leaves of the tree in DT [13]. Predictions from DTs can exhibit significant variability. Minor alterations in data may lead to disparate outcomes [14]. The DT approach has been employed to create combinatorial libraries, forecast drug-likeness, anticipate specific biological activities, and produce compound profiling data. DTs can forecast ADME/Tox characteristics, encompassing drug absorption, distribution, solubility, permeability, P-glycoprotein or blood–brain barrier penetration, and metabolic stability [22].

### 3.3. RF

An ensemble classifier made up of several DTs is called RF [22]. This concept refers to a method of generating a forest from multiple viewpoints to achieve randomness [24]. The ability of this algorithm to model intricate data and produce accurate predictions sets it apart. The algorithm works by producing a large number of DTs and combining their predictions [13]. The various DTs are trained using the provided features and a random subset of the input data. This stochastic approach enhances the final model's generalization performance and decreases overfitting [25]. Random sampling is employed to create a training set for each tree, substituting the original dataset. In the process of tree construction, one-third of the occurrences are excluded and employed as a test set. The categorization performance of the test set is evaluated by out-of-bag error rates [22]. RF is frequently employed in pharmaceutical research for disease classification, quantitative structure-activity relationship (QSAR) modeling, and forecasting ligand–protein binding affinity. It facilitates the study of patient data to optimize treatments and enhances docking precision by refining scoring systems. RF-based QSAR models can forecast the efficacy and safety of medications by analyzing molecular attributes. Notwithstanding constraints in chemical structure modeling, RF continues to be an efficacious method in computational drug development [24].

### 3.4. Naive Bayesian Classifier

A probabilistic technique called Naive Bayesian determines whether a given input is likely to belong to a class by using previous probabilities and distributions [26]. This strategy accepts that all qualities are independent of each other. Naive Bayes is particularly advantageous when the dataset is sparse and numerous input features are present. A labeled dataset is employed to train the classifier, which utilizes the training data to ascertain the prior and conditional probabilities. Naive Bayes classifiers are classified into three types: “Gaussian, Bernoulli, and Multinomial Naive Bayes.” The Gaussian Naive Bayes assumes that the input features follow a Gaussian distribution. The Bernoulli Naive Bayes classifier is employed for binary input features, indicating the presence or absence of an attribute, whereas the Multinomial Naive Bayes classifier is utilized for discrete count input features [13]. This is employed to categorize the bioactivity of drug-like compounds, predict the toxicity of the chemical, and discover protein targets [14].

### 3.5. CNN

CNNs are specialized neural network architectures that use four basic components to interpret information, such as “a convolutional layer, a rectified linear unit, a pooling layer, and a fully connected layer” [13]. CNNs are a subset of DL that develop the ability to differentiate between various kinds by processing inputs and assigning weights to certain elements of the input (AYENI, 2022). CNNs are adept at analyzing spatial data, rendering them ideal for predicting molecular characteristics from chemical structures and evaluating high-content screening images. CNNs are an effective approach for identifying physiologically relevant patterns, as they can autonomously extract hierarchical features from input data, minimizing the need for extensive human feature engineering [13]. CNN models are utilized in diverse research, including image analysis for predicting cardiac failures, QSAR models, digital pathology biopsies, and protein–ligand affinities [24]. Various sophisticated DL models utilizing CNN, including “AlexNet, Xception, Inception, Visual Geometry Group (VGG), and ResNet,” can be employed in the domain to investigate conceptual biological discoveries [13].

### 3.6. RNN

A RNN is a type of neural network that employs a self-learning mechanism through the formation of hidden layers and generative input processing. Connections may exist between the input and output nodes [27]. The RNN architecture facilitates the cyclical flow of information through the arrangement of multiple interconnected nodes in a directed loop [13]. This facilitates the formation of a temporal sequence and a directed graph within the network. The RNN network utilizes internal memory to classify input variables [24]. To address complex challenges, versions of RNN, including “Long Short-Term Memory and Gated Recurrent Unit,” were developed to lessen the vanishing gradient problem inherent in basic RNNs [13].

### 3.7. GNN

A model designed for DL that processes graph-structured data is referred to as a GNN [28]. GNNs disseminate information from neighboring nodes to progressively refine the attributes of nodes within a graph. The main structural trait of graphs is that nodes need not be arranged in a certain order, and any functions applied to them should exhibit permutation invariance, or independence from order. The output of these functions remains consistent for any two isomorphic graphs. A standard GNN comprises one or more layers that execute a node-wise aggregation from neighboring nodes; this aggregation must be permutation invariant due to the variability in neighbor ordering [29]. GNNs are exceptionally adept at modeling data represented as graphs, such as networks of protein–protein interactions, chemical structure, and molecular and genetic data, which are computationally analyzed in drug development to anticipate molecular activity, find possible therapeutic targets, and investigate connections within biological processes [13].

### 3.8. GAN

A distinctive and progressive neural network architecture called a GAN entails the simultaneous training of two networks, one focused on detection and the other on image generation. To produce novel instances, the generator typically captures the distribution of authentic cases. A binary classifier functions as the discriminator, effectively distinguishing between generated instances and authentic examples [30]. When the discriminative network module identifies an event as more probable, it predicts a higher likelihood. Simultaneously, the generative network module is trained to enhance the likelihood of the discriminative network module committing an error. Both the generative and discriminative networks concurrently compete to attain their objectives. The GAN architecture facilitates an adversarial interaction between the generative and discriminative network components. It possesses significant potential for use across various domains, including drug research and discovery, photography, videography, linguistics, and more [31]. The ReLeaSE program utilizes a GAN5 to suggest synthetic pathways for target molecules based on a repository of established reactions [13]. Additionally, MolGAN and ORGAN are two straightforward models incorporated into the GAN framework for drug likeliness and synthesizability [28].

### 3.9. BERT

BERT is a transformer-based DL model that is among the most extensively utilized DL language models. It utilizes bidirectional self-attention by simultaneously conditioning the left and right contexts. Two unsupervised jobs, that is, masked language modeling and next sentence prediction, can be employed to pretrain BERT. The MLM aims to predict the masked words only based on their context, which involves randomly obscuring a segment of the input sequence's tokens. A sequence is forecasted from the prior sequence utilizing the BERT model, which has experienced pretraining on text pairing representations for the NSP task. Furthermore, BERT has undergone pretraining in computational biology, bioinformatics, and drug discovery [32]. BERT models have various applications, including sequence analysis, genome analysis, gene expression, proteomics, and biomedical informatics [20]. Diverse transformer models are employed in molecular property prediction and drug development, including SMILES-BERT, ChemBERTa, and K-BERT. SMILES-BERT is utilized for molecular property prediction by fine-tuning, ChemBERTa is applied for multimodal visualization and downstream performance, and K-BERT is employed to acquire atomic information and forecast molecular characteristics. Furthermore, DNABERT is employed for the prediction of promoters and the identification of transcription factor binding sites.

## 4. Metrics for Evaluating ML Models in Drug Discovery

Assessing a model is essential for doing adequate validation. Evaluation serves two purposes: promising ideas can be refined, while ineffective ones can be discarded. Moreover, it is often beneficial to assess if an ML model outperforms an expert, especially in the medical domain [33]. SML entails partitioning data into training and test sets, training and verifying the model using the training data, generating predictions for each instance in the test data, and comparing these predictions to the matching ground-truth values in the test set. This enables us to ascertain whether the predictions of a new ML model surpass those of humans or existing models in our test set [34]. Despite the significance of evaluating an ML model, novice researchers, whether intentionally or unintentionally, misread the performance of the trained models, resulting in erroneous predictions. A comprehensive research by Rainio and coworker, demonstrated the importance of statistical tests and assessment measures in ML [35]. We have made a comparable effort in the Table, where commonly utilized metrics for assessing classification and regression types of ML models in drug discovery are enumerated.

Consequently, we successfully organized many metric names, their descriptions, formulas, applications, value ranges, optimal values, and interpretations in Table 1. A robust performance model will exhibit values approaching 1 for metrics like “Accuracy, F1, AUC, and *R*^2^,” while error-based metrics like “MAE, MSE, RMSE, and MAPE” should yield values close to 0. A negative *R*^2^ or an Matthews's correlation coefficient (MCC) around 0 indicates that the model may underperform compared to random chance, while a ROC–AUC of 0.5 suggests that the model is making arbitrary guesses. In the event of an imbalanced input dataset, the critical metrics are “balanced accuracy, F1-score, MCC, and precision–recall curve area under the curve (PRC–AUC).” This will enable us to enhance predictive accuracy and optimize model performance.

## 5. Handling Imbalanced Data Sets

The accessible drug development databases often contain hundreds or thousands of times more inactive compounds than active ones, highlighting a significant imbalance [36]. Forecasting the minority-interested class is a rare event; disregarding it entirely or categorizing it as an anomaly or noise results in bias and suboptimal generalization performance [37]. Although numerous prior studies in the fields of illness and pharmacology indicated that effectively correcting class imbalance would improve model performance [38, 39]. Imbalanced data are a significant challenge in ML models, frequently affecting their performance and accuracy. A variety of solutions have been devised to efficiently solve this issue. ML is extensively utilized in biological sciences, improving the efficiency and accuracy of research and analysis. The tactics outlined in this section are commonly employed.

### 5.1. Data-Level Techniques

The initial approach for addressing unbalanced data involves data-level approaches. The data may be subjected to either oversampling or undersampling. Oversampling is a method employed to augment the data of the minority class, whereas undersampling is utilized to reduce the data of the majority class.

#### 5.1.1. Oversampling

Random oversampling entails the random selection and duplication of samples from the minority class to generate extra examples. This method equilibrates the dataset, enabling the model to learn from both classes more efficiently during training [40]. The Synthetic Minority Oversampling Technique (SMOTE) is a prevalent random oversampling method aimed at mitigating class imbalance through the generation of synthetic data. It improves dataset equilibrium by incorporating fresh samples instead of simply replicating existing ones. SMOTE applies to both extensive and limited datasets, including gene expression data, providing significant theoretical insights and empirical findings [41]. Adaptive Synthetic Sampling (ADASYN) is a sophisticated oversampling method derived from SMOTE. Similar to SMOTE, it produces synthetic data to assist ML models in learning from imbalanced datasets. In contrast to SMOTE, ADASYN emphasizes the more difficult minority class situations, hence enhancing representation and facilitating superior learning for intricate decision boundaries [42]. Moreover, Borderline-SMOTE is an advanced iteration of SMOTE that produces synthetic instances in proximity to the decision boundary separating classes. This method enhances the management of imbalanced datasets by concentrating on pivotal areas where misclassification is more probable. The Borderline-SMOTE is frequently integrated with the Artificial Immune Recognition System (AIRS) for comprehensive optimization, whilst the nearest neighbor method functions as a local classifier to enhance the learning process [43].

#### 5.1.2. Under-Sampling

Random under-sampling entails the selection of a random subset of instances from the majority class, with the exclusion of the remaining instances. This facilitates the attainment of an equitable class distribution according to a predetermined ratio. The subset size is dictated by the intended class balance, guaranteeing that the model is trained on a more representative dataset [44]. This technique employs the NearMiss-1 method, which identifies the majority class samples that closely resemble several minority class examples. Selection is determined by the minimal average distance to the three closest minority class samples, hence enhancing class equilibrium in the dataset [45]. Alternatively, Tomek links (T-links), created by Ivan Tomek, are a sophisticated undersampling method aimed at mitigating imbalanced datasets by diminishing noise. This technique discerns and eliminates instances from the majority class that are nearest to the minority class. T-links comprise paired instances from contrasting classes that are nearest neighbors. The strategy promotes data clarity and improves classification performance by eliminating these marginal instances [44]. Moreover, the One-Sided Selection approach, proposed by Kubat [46], is an undersampling technique that integrates T-link and Condensed Nearest Neighbor algorithms to preserve class imbalance in datasets. It eliminates instances from the majority class deemed redundant [47]. Finally, the cluster-based undersampling method employing centroids preserves class equilibrium in datasets by leveraging the K-means clustering technique to substitute majority class samples with their respective centroids. Furthermore, it utilizes K-means clustering to detect and eliminate instances of the majority class according to their proximity to the cluster centroids, so successfully minimizing redundancy while maintaining data integrity [48].

### 5.2. Data Augmentation Techniques

Data augmentation is a method employed to enhance the size of training datasets by producing synthetic data to preserve balance. Utilizing sophisticated ML models, like “Variational Autoencoders (VAEs) and GANs,” enables the generation of novel, realistic data points through the understanding of diverse data distributions. GANs use an unsupervised learning approach to analyze and learn from data, generating new samples. Autoencoder neural networks comprise two deep learning components: an encoder and a decoder. The encoder facilitates the conversion of input data from their original form, allowing the decoder to recreate the data [49, 50].

### 5.3. Algorithm-Level Techniques

Another method to address data imbalance involves algorithmic-level strategies that employ classifiers. Classifiers employed in algorithmic-level methodologies are less influenced by class imbalance phenomena [51, 52]. Nevertheless, it is imperative to design approaches that address uneven data and minimize classification costs. These methodologies encompass cost-sensitive procedures, ensemble techniques, and boosting strategies, among others.

#### 5.3.1. Cost-Sensitive Learning

Cost-sensitive learning tackles the problem of data imbalance. This strategy incorporates miscalculation costs for the minority class to guarantee the model prioritizes it and maintains equilibrium during training [53, 54]. A fundamental illustration of cost-sensitive learning is weighted loss functions. It aids in adjusting the loss occurrences within the minority classes. This is typically employed when a moderate class imbalance exists [31]. XGBoost with scale_pos_weight is another instance frequently employed to rectify class imbalance by modifying the weight distribution between positive and negative classes. The scale_pos_weight option enhances the significance of the minority class, hence augmenting classification performance. For instance, if the model frequently misclassifies the minority class, augmenting the scale_pos_weight value prompts the algorithm to predict the minority class with greater precision [55].

#### 5.3.2. Ensemble Learning

For an algorithmic-level technique, Easy Ensemble is a prominent subtype. It is a straightforward technique that involves training the ensemble classifiers on random undersampling datasets and combining the predictions to enhance the imbalanced datasets. Easy Ensemble and Balanced Random Forests both try to use balanced bootstrap samples. Balance Cascade is another Ensemble learning method that is quite similar to Easy Ensemble. For each repetition, it creates new balanced training subsets by replacing the correctly classified subsets. This helps to maintain the training data balance. Widely used in areas such as medical diagnoses or fraud detection [56, 57].

#### 5.3.3. Boosting Methods

Boosting approaches represent the third category of algorithmic techniques for addressing imbalanced datasets. A variant of the boosting technique is Adaptive Boosting (AdaBoost). It is an ML algorithm that integrates several weak classifiers to create a more robust classifier. It modifies the weight of the training instances according to the errors committed by the classifier in prior iterations. Initially, it equilibrates the weights, trains the weak classifier, and assesses its efficacy. The weight may be adjusted according to the errors committed by the poor classifier. For example, AdaBoost's principal characteristics include dynamic weight adjustment and the absence of prerequisite knowledge [58]. RUSBoost, a variant of boosting algorithms, was introduced by Seiffert by random undersampling. It integrates random undersampling and boosting to enhance the efficacy of imbalanced datasets. RUS functions akin to undersampling by eliminating instances from the majority class to balance the dataset, followed by the use of boosting. RUSBoost is built from the SMOTEBoost algorithm [59, 60]. SMOTEBoost is a consolidation of SMOTE and boosting, created by Chawla and colleagues [61]. SMOTEBoost produces synthetic instances for each iteration of the minority class, hence improving model accuracy for that class. It modifies weight distributions, trains weak learners utilizing CART-based DTs, and enhances model robustness in imbalanced settings [60, 61]. Xtreme Gradient Boosting (XGBoost) is a robust tree-based boosting method optimized for extensive datasets. It enhances weak classifiers, primarily DTs, to construct a resilient model. XGBoost adeptly manages sparse features and absent values, guaranteeing superior performance despite inadequate data [62].

### 5.4. Hybrid Approaches

In rare cases, where none of the said strategies effectively address an imbalanced dataset, hybrid approaches may be the sole remaining choice. These are extensively utilized in ML to preserve equilibrium within the input data. SMOTE combined with cost-sensitive learning is a hybrid approach designed to preserve class balance, wherein SMOTE produces synthetic samples for the minority class, while cost-sensitive learning assigns elevated misclassification costs to the minority class, thereby addressing data imbalance [63]. A combination methodology utilizing SMOTE and Ensemble may train several classifiers and amalgamate their predictions, hence enhancing classification performance through ensemble learning and the incorporation of synthetic cases [64]. Several sophisticated techniques exist to address the imbalanced dataset issue. Self-supervision is especially advantageous in low-data contexts, as it is independent of external data sources. This method aids in preserving equilibrium within the datasets. It is utilized when the dataset is limited, without dependence on extensive external datasets.

## 6. Emerging Trends in AI for Drug Discovery and the Prominently Used AI-Enabled Tools

**Figure 2:**
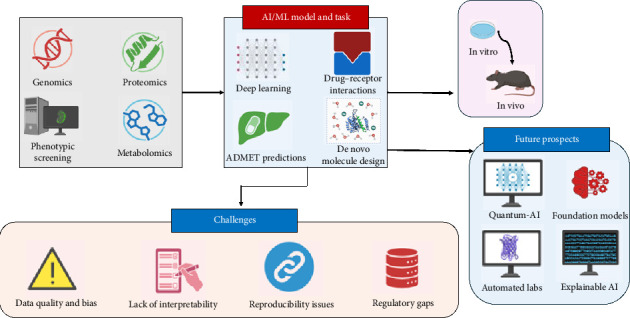
Progress of AI and ML in drug discovery.

In recent years, AI has gained prominence in the biomedical sector due to its capability to analyze and understand extensive datasets that conventional statistical methods cannot manage [65]. Many traditional studies also signify the importance of MIA-QSAR and Aug-MIA-QSAR in the drug discovery process [66, 67]. Despite the successful creation and licensure of new pharmaceuticals showcasing the efficacy of conventional methods in drug discovery and early clinical development, there remains significant potential to utilize AI to enhance existing models. Moreover, AI presents the opportunity to develop innovative solutions that might expedite and transform these processes [68]. Furthermore, it can assist with drug activity, protein–protein interactions, de novo drug design, and the three-dimensional structure prediction of a specific protein [69]. The progress of AI and ML in drug discovery is illustrated in Figure 2.

### 6.1. ML

A subset of AI known as ML enables the analysis of intricate biological data, including genetic information, chemical structures, and disease models, to identify potential medication candidates more swiftly and accurately than traditional methods. Their utilization in drug repurposing, which identifies new applications for existing approved treatments, offers potential for neglected diseases and reduces the time and cost associated with drug development [70, 71]. To conduct this inquiry, we must effectively train a resilient model and validate its predictions as indicated in the preceding section.  Table 2 summarizes commercially and noncommercially available tools for training and constructing ML models. This information will enable researchers to locate multiple tools in one location according to their specific interests.

### 6.2. De Novo Drug Design and Molecular Generation

The vast “chemical universe,” estimated to encompass up to 10^60^ minuscule molecular entities, is a significant challenge for researchers [72]. Identifying novel chemicals with distinct characteristics and efficiently investigating such an extensive area are, therefore, exceedingly challenging endeavors. A computer-assisted technique, De novo drug design, can generate novel molecular structures from atomic components without any prior connections. This approach can directly facilitate the exploration of an extensive chemical space [30]. AI and other DL techniques can serve as a novel resurgence in de novo design. The following section enumerates the predominantly utilized AI-enabled tools that facilitate progress in De novo drug design.

REINVENT is a readymade tool for de novo design, with the source accessible at (https://github.com/MolecularAI/Reinvent) on GitHub. This aids drug discovery by creating molecules with RNNs, facilitating the identification of novel drug-like compounds. It provides two scoring functions, with the cumulative score varying from 0 to 1. It facilitates a versatile approach for formulating intricate MPO scores and can be utilized for both exploration and exploitation [73].

MOLGPT is a transformer-decoder model that autonomously creates SMILES strings to create compounds with specified attributes. MOLGPT functions as a little variant of the GPT model, possessing merely 6 million parameters. MOLGPT preserves the scaffold, TPSA, and logP values while enhancing the SAS and QED values of an initial molecule [74].

The Junction Tree Variational Autoencoder (JT-VAE) is a graph-based VAE. It outperforms the diverse models in molecular reconstruction, creates a tree of chemical substructures, and integrates these substructures into a molecular graph. It enhances chemical validity, latent space, and property optimization [75].

ChemTS is an innovative Python module that offers a versatile de novo molecular design tool. It is an RNN integrated with Monte Carlo tree search (MCTS) and trained on extensive SMILE datasets, demonstrating superior performance compared to other libraries such as VAE in producing novel compounds and improving attributes like absorption [76].

### 6.3. Molecular Language Models

Besides the AI-enabled De novo application, AI can also facilitate the comprehension of the molecular features of chemical compounds. Below are samples that summarize the prevailing language models employed to analyze chemical structures.

MolBERT is a transformer-based pretrained language model comprising three components: a feature extractor, pretraining, and fine-tuning. SMILES, the featurizer, adapts molecular representations and transmits them to the feature extractor, with the outputs then relayed to the BERT module [77].

ChemBERTa is a transformer-based language model that has been utilized by RoBERTa. It is optimal for toxicity prediction, solubility estimation, and bioactivity classification. ChemBERTa demonstrates superior downstream performance on MoleculeNet and effective attention-based visualization methods, improving with the size of the pretraining dataset [78].

The SMILES Transformer is an encoder–decoder model based on the transformer architecture. It differs from RNN; it is more rapid and stable. The model is trained using 861,000 unlabeled SMILES randomly selected from ChEMBL24. Facilitates QSAR modeling by developing models of drug-like compounds [79].

### 6.4. Protein Structure Prediction and Drug-Target Interaction

The extensive months to years of diligent effort required to ascertain a single protein structure constrain structural coverage and to create a balance between known protein structures and known protein sequences. Large-scale structural bioinformatics and precision computational approaches are significantly contributing to bridging this gap. The significance and assistance of AI in protein structure prediction became evident after the advent of AlphaFold and the subsequent awarding of the Nobel Prize, following extensive endeavors [80]. This section offers a concise overview of analogous tools.

AlphaFold2 is a DL model designed for predicting three-dimensional protein structures. It demonstrated enhanced accuracy in predicting structures during CASP14. AlphaFold2 was developed to correlate amino acids and forecast the three-dimensional structures of proteins, offering predicted Local Distance Difference Test (pLDDT) scores to indicate the reliability of its predictions [80]. Recently, AlphaFold3 has been introduced with enhanced functionalities.

RoseTTAFold is a program that employs DL to predict three-dimensional protein structures, akin to AlphaFold2, and was developed by the Baker laboratory. RoseTTAFold program utilizes a three-track neural network to predict diverse structures with limited available data, generating monomeric, multimeric, and protein–protein complex structures [81].

DeepPurpose is a DL library utilized for predicting DTI, which is essential for drug discovery. It employs the encoder–decoder framework for DTI predictions, distinguishing it from other libraries [82].

### 6.5. Virtual Screening and Docking

Molecular docking, another computational advancement, predicts the binding affinity of ligands to receptor proteins. In recent years, molecular docking, enhanced by advanced AI applications, has become an essential element of in silico drug development [83]. A variety of computational tools and approaches, both free and commercial, exist for molecular docking procedures. This section discusses some entities that expressly employ AI.

Deep Dock is a universal neural network and an innovative method for virtual screening, as opposed to conventional techniques. It is utilized for docking investigations and analyzing protein–ligand interactions by comprehending the structures of both the ligand and the protein [84].

Gnina is an open-source docking software that employs DL for predictive analysis. It includes the protein, ligand structures, and the designated binding site. The docking procedure explores the conformational space of the ligand by Monte Carlo sampling, with evaluations expressed as RMSD scores [85].

AutoDockFR (ADFR) is an open-source software built inside the AutoDock suite. AutoDock-FR will considerably broaden the range of biological problems addressed by automated docking, offering the ability to incorporate new methods and uniquely integrating ML for output, including docking poses [86].

EquiBind is a novel AI-driven molecular docking tool. This tool is an SE(3)-equivariant geometric DL model capable of directly predicting the ligand's bound conformation and orientation, as well as the receptor binding site (blind docking) [87]. TANKBind is a contemporary AI-driven molecular docking tool. TANKBind incorporates trigonometric restrictions as a robust inductive bias, directly addressing each possible binding site for every protein by segmenting the entire protein into functional chunks [88].

### 6.6. Graph-Based Learning (GNNs) Tools for Chemical Structure Analysis

GNN-based chemical structure analysis tools are also in demand because of their robust and reliable predictions. For instance, DGL-Lifesci (Deep Graph Library-Lifesci) is an open-source, free tool for generating graph data using DL methods. DGL-Lifesci provides high-quality and robust 7 GNN-based models for molecular property prediction, reaction prediction, and molecule generation. It was developed using RDKit, PyTorch, and DGL. Additionally, this tool also has the advantage of hyperparameter adjusting, rather than just using the default parameters [89].

Chemprop is the tool that is used for the prediction of chemical properties by using the directed message-passing neural networks (D-MPNNs). It makes D-MPNNs train out of the box and makes it faster, simpler, and opensource for the predictions. It predicts the properties for various systems that contain molecules like solute, solvent, combinations, or reactions with or without solvent [90].

Attentive FP is another GNN that uses a graph attention mechanism for molecular representation. According to attentive FP, it can automatically learn nonlocal intramolecular interactions from given tasks, allowing us to immediately derive chemical insights from data that are not visible to the human eye [91].

Last but not least, MolGraph is a Python package that is used for implementing molecular graphs and GNNs using TensorFlow and Keras. It is mainly used in ML for the prediction of molecular properties and molecular identification, and it is an open-source, free tool [92].

### 6.7. Multiomics and Systems Biology

The subject of integrative genetics evolved because of the introduction of genotyping arrays, which made it possible to perform substantial genome-wide association studies and tools for looking at transcript levels globally [93]. These days, biological researchers commonly combine other omics technologies, such as proteomics and metabolomics, into their daily workflow. Various tools expressly deploy the usage of AI for faster and reliable outcomes. For example, Specific cOntext Pattern Highlighting In Expression (SOPHIE), a multiomics AI tool, uses a chosen template experiment to discern between common and transcriptional signals using a generative neural network to imitate a series of background transcriptome experiments. Specifically, the log2 fold change data provided by standard DE investigations can be augmented by SOPHIE's specificity measure [94].

Another AI-enabled multiomics tool is DGMP. DGMP integrates the directed graph convolutional network and multilayer perceptron to identify cancer driver genes with multiomics pan-cancer data, including: “gene expression, mutation, copy number variation, and DNA methylation” [95].

Likewise, scEMAIL is an AI-driven multiomics tool that serves as a comprehensive annotation framework for scRNA-seq data. Novel cell types can be readily identified without accessing the original material during adaptation [96].

Multiomics Factor Analysis (MOFA+) is intended for the thorough integration of multi-modal single-cell data. It is mostly utilized for unsupervised learning to manage specified labels, specifically engineered for processing larger datasets. The software integrates transcriptomics, epigenomics, proteomics, and metabolomics [97].

DeepMOCCA (Deep Multiomics Cancer Analysis) is a DL program designed for predicting tumor characteristics via graph attention mechanisms. It anticipates characteristics at the molecular level and functions as an end-to-end learning model that generates representations of nodes and cancer samples, predicting survival based on multiomics cancer data [98].

### 6.8. Clinical Trial Applications

AI-enabled technologies and real-world data, or scientific data from several sources, have begun to change how we approach clinical trials. This allows us to reconfigure important aspects of clinical trial design through AI approaches [99, 100]. Numerous earlier studies have demonstrated that AI-based clinical trial matching algorithms enable very accurate and efficient screening of cancer patients for clinical trials [101, 102].

AI also facilitates the anticipation of medication safety by combining clinical, biological, and chemical data to predict adverse drug reactions (ADRs), thereby reducing the number of late-stage trial failures. Early risk mitigation is made possible by the efficacy of DL models in predicting hepatotoxicity and cardiotoxicity [103]. Through the use of EHRs, AI-driven clinical trials improve personalized treatment by identifying appropriate patient populations based on clinical, genetic, and molecular traits. By facilitating accurate patient enrollment, flexible trial design, and customized treatment plans, this integration enhances therapeutic results and speeds up drug development [104].

Furthermore, AI has the potential to improve participant diversity, lower sample size requirements, and influence clinical trial eligibility criteria. Trial Pathfinder, an open-source AI tool created by Liu and their team, simulated clinical trials using electronic health record (EHR) data by integrating EHR data based on various inclusion criteria and calculating the overall survival risk ratio, which is the difference in survival rates between two or more patient groups [105].

## 7. Limitations and Challenges of AI in Drug Discovery

Although AI has potential for drug development, some important restrictions and hurdles need to be carefully considered. The availability of appropriate data is one of the main obstacles. Large datasets are typically necessary for AI-driven methods to learn effectively, especially if they are not of optimal quality. Large, varied, high-quality datasets are essential for AI models, but biological data are frequently fragmented, heterogeneous, and biased toward certain populations or illnesses, leading to overfitting, bias, and poor generalizability [106]. Additionally, the lack of qualified workers to run AI-based platforms, small businesses, tight budgets, concerns about replacing humans and losing jobs, skepticism about AI-generated data, and the “black box” phenomenon (the process by which the AI platform draws its conclusions) are additional obstacles to the full adoption of AI in drug discovery [107]. The use of AI in drug development also has several drawbacks and difficulties, which are discussed below:

### 7.1. Data Availability and Quality Issues

The main challenge with using AI to discover new drugs is the difficulty of obtaining high-quality, properly labeled files. Large, diverse, and representative datasets are crucial for training and validating DL models. Often, biomedical data are inconsistent, incomplete, and varies widely. This data originates from diverse sources, like chemical assays, genomics, and clinical phenotypes. These sources do not always follow standard formats, units, or metadata, complicating the process of combining them into unified training datasets [108]. Important metadata, including cell line information, experimental batch, measurement timing, and assay conditions, is not always recorded accurately, which hampers reproducibility and conflicts with the “FAIR (Findable, Accessible, Interoperable, Reusable)” data principles. Additionally, the data can be biased and inaccurate. Many datasets are skewed, often due to an excess of positive bioactivities or specific chemical scaffolds and populations. This bias can hinder model generalization and lead to overconfidence in incorrect predictions [109]. When applied to larger, more diverse patient populations or in unfamiliar chemical domains, models developed from limited geographical or demographic data often perform poorly. Moreover, there is a scarcity of balanced, well-labeled datasets, especially for rare diseases or targets that are difficult to identify. While public databases like ChEMBL are carefully curated, they may not always contain high-confidence bioactivity data labeled consistently. The absence of negative data further diminishes their usefulness [110]. These issues, combined with small sample sizes and high-dimensional data, increase the risk of overfitting and reduce data reliability. Benchmarking and reproducibility pose additional problems. Many ML models are tested on outdated datasets that tend to overestimate real-world performance. Prospective validation is often missing, and problems such as label leakage and unrealistic benchmarks are common. Reproducibility issues in preclinical research mean that studies published in high-impact journals are often irreproducible, raising concerns about the reliability of training data [111].

Various strategies are discussed in the literature to address these challenges. Thorough data curation, including detailed metadata with specific experimental conditions and quality control metrics, is essential for identifying biases and ensuring trust in similarities across studies [111]. Data augmentation techniques such as synthetic data generation, transfer learning, active learning, multitask learning, domain adaptation, and FL help address data fragmentation and scarcity [112]. Methods for bias reduction, including SMOTE for oversampling and robust uncertainty quantification, improve model calibration and reduce overfitting risks. Ultimately, comprehensive prospective evaluation in real-world scenarios is vital to confirm that models are applicable beyond the training context. If these validations fail, the training procedures should be revisited [112, 113].

### 7.2. Bias and Interpretability in ML Models

Use of ML in drug research is restricted by problems with bias and interpretation. Bias often happens because datasets are skewed or not representative, like having too many of certain chemical scaffolds or not enough population diversity. This leads to predictions that cannot be applied to other situations and could be dangerous [10, 114]. Also, complicated models like deep neural networks often work like “black boxes,” making it harder to see how they make decisions. This makes it harder to trust them in important areas like predicting danger or effectiveness [114].

For validating forecasts and getting mechanistic insights, interpretability is a must. Still, many effective models put more emphasis on correctness than on being able to explain things. Methods like SHAP, LIME, and attention mechanisms can explain things after the fact, but they often cannot come up with biologically important meanings [115]. Fairness and readability can be improved by using transparent models, putting humans in the loop, and incorporating domain knowledge [116]. This makes AI use in drug discovery safer and more accountable.

### 7.3. Chemical Diversities

Achieving sufficient chemical diversity in training, screening, and generating datasets is one of the main obstacles to using AI/ML for drug development [117]. Generative approaches frequently yield molecules skewed toward known scaffolds or straightforward modifications of them, and models trained on limited chemical scaffolds frequently fail to generalize to novel chemotypes [118]. When the objective is scaffold hopping or novel chemical entities, this becomes more difficult [117, 119]. To tackle this issue, several approaches have been investigated, including the use of explicit diversity-driven terms in generative model reward functions (e.g., through RL with exploration strategies) [120], the creation of synthetic libraries to cover underrepresented scaffolds [121], and the use of coarse-grained chemical space sampling [122], and so on.

A well-known model, DrugEx, uses exploration tactics to improve chemical diversity for the adenosine A_2_A receptor [120]. Furthermore, in other examples, the complexity-to-diversity and pseudo-natural product synthetic techniques have been used to generate compounds with skeletal and stereochemical novelty [123]. According to Elton and their team, these methods can increase the probability that leads generated by AI/ML will become viable drug candidates by reducing the overfitting and limited domain coverage that are common in many QSAR or generative pipelines [119].

### 7.4. Reproducibility

Reproducibility continues to be a significant hurdle in AI-driven drug discovery. Numerous ML models are irreproducible owing to inadequate data sharing, the absence of defined workflows, and insufficient documentation of hyperparameters and model configurations. These difficulties result in inflated performance assessments and impede scientific advancement. Methodological issues, including data leakage, selective reporting, and opaque preparation, are commonly encountered. The lack of prospective validation restricts the practical usability of numerous AI models [11, 124].

To tackle these difficulties, experts have suggested optimal techniques such as employing containerized environments, ensuring transparent model documentation, and standardizing workflows. Community-wide initiatives and reproducibility frameworks are emerging to foster rigorous validation and open science practices. Reproducibility is crucial for both scientific credibility and regulatory approval of AI technologies in drug development [125].

### 7.5. Ethical and Regulatory Considerations

Ethical and legal issues have received significant attention as AI is increasingly used in medical research. The main concerns include data privacy, informed consent, algorithmic bias, and the lack of transparency in AI-driven decision-making. These issues raise questions about who is responsible when AI predicts medication effectiveness, safety, or patient reactions [126]. Biased training data can perpetuate health disparities, and the complexity of many DL models hampers understanding, undermining trust and complicating regulatory approval [127]. Currently, there are no standard methods to evaluate AI systems used in biomedical contexts. The FDA and EMA's approval processes are not yet equipped to assess dynamic, self-learning systems [128]. Moreover, AI technologies must be interpretable and verifiable to meet standards of public trust and clinical accountability [129]. For AI to be effectively integrated into pharmaceutical research, it must be fair, transparent, and adhere to ethical regulations like the EU's General Data Protection Regulation (GDPR).

### 7.6. Bridging the Gap Between Computational Predictions and Experimental Validation

AI and computational models have enhanced early-stage drug discovery via virtual screening, target identification, and activity prediction; however, a significant challenge remains in converting these predictions into experimentally validated results. Numerous silico results do not translate successfully to wet-lab environments, attributed to oversimplified models, insufficient biological context, or erroneous assumptions regarding target-ligand interactions [130]. Discrepancies between training data and real-world biological variability may result in false positives or irreproducible outcomes [131].

Integrated workflows that combine AI-based predictions with high-throughput in vitro and in vivo assays are essential to address this gap. Iterative feedback loops, in which experimental data refine model parameters, enhance prediction accuracy and biological relevance. Furthermore, effective collaboration between computational scientists and experimental pharmacologists is essential to ensure that models are developed with translational applicability as a priority. Experimental validation is the definitive standard, and its congruence with computational insights is essential for progressing AI-driven drug development pipelines [132].

### 7.7. Clinical Translation

Despite significant advancements in AI-enabled clinical trials, the clinical translation of computational predictions remains another significant barrier in AI-driven drug development. Due to the biological, pharmacological, and therapeutic complexity of human systems, many AI-identified hits do not effectively advance beyond preclinical and clinical phases [133]. Human pharmacokinetic (PK), pharmacodynamic (PD), and toxicity characteristics are frequently missed by models that were mainly trained on in vitro or preclinical datasets [103]. Additionally, the credibility of AI-based outcome predictions is diminished by data heterogeneity, a lack of clinical annotations, and variations in patient demographics among research [134].

As a solution, one can integrate multimodal datasets, such as genetic, clinical, PK, and PD data, which helps improve model generalizability and translational reliability in AI-enabled clinical trials. Interpretability and clinical trust are enhanced by using XAI techniques and continuously retraining models using real-world clinical data [104].

## 8. Future Directions of AI in Drug Discovery

The future of AI in drug discovery is set for significant advancements through the integration of multimodal data, enhanced model interpretability, and increased collaboration between computational and experimental fields. A number of new computational paradigms, including FL, XAI, and quantum computing, have the potential to revolutionize precision drug discovery and development, while their timeframes for implementation differ significantly. FL and privacy-preserving AI facilitate collaboration among institutions while maintaining patient confidentiality, thereby enhancing access to underutilized datasets [135]. FL can create reliable models for patient classification and biomarker identification in drug discovery by combining various chemical and biological information while maintaining privacy. Within a few years, FL is anticipated to attain near-term translational application due to the growing acceptance of multi-institutional frameworks [136, 137]. However, data format variability and non-independent and identically distributed (non-IID) distributions across locations are examples of bottlenecks in the case of FL.

Similarly, AI models created for interpretability and transparency, and to avoid the black box phenomenon of AI, are referred to as XAI. XAI assures expert-domain users to understand and believe in the predictions [138]. XAI supports regulatory compliance, forecasts off-target effects, and clarifies mechanisms of action in drug research. Translational preparedness in the near future is suggested by the growing integration of techniques like SHAP and LIME into predictive processes [139]. Developing consistent evaluation measures and striking a balance between interpretability and model accuracy are the challenges in the field of XAI. Also, it is acknowledged that XAI is in its infancy. It will take some time to come to its peak.

Unlike conventional computers, quantum computing processes complicated molecular interactions more quickly by utilizing quantum mechanical phenomena like superposition and entanglement. Improved virtual screening of chemical libraries and more precise predictions of DTIs have been made possible by the combination of quantum computing and ML techniques [140]. The integration of AI with quantum computing, systems pharmacology, and generative models such as diffusion and transformer-based architectures has the potential to yield novel drug-like molecules with enhanced precision [141, 142]. Several obstacles still exist, though, including legal concerns, skilled staff shortages, algorithmic maturity, and hardware restrictions in drug discovery through quantum mechanics [140].

Recent developments in self-supervised and foundation models, which can learn from a variety of biological, chemical, and clinical data, are anticipated to enhance hit identification, ADMET prediction, and personalized drug development [143]. Future research must prioritize standardization, benchmarking, and reproducibility to guarantee that AI tools are robust, interpretable, and applicable in clinical settings. Cross-disciplinary collaboration is essential for fully harnessing AI's potential in both preclinical and clinical drug development phases.

## 9. Conclusion

This review examines the significant influence of AI and ML on the evolution of drug discovery. Various ML models have been discussed, including “supervised, unsupervised, semisupervised, reinforcement learning, and deep learning” architectures. Each model contributes uniquely to key stages of the drug development pipeline, such as “target identification, virtual screening, de novo drug design, and ADMET prediction.” We examined prevalent ML algorithms, validation metrics, and AI tools that enhance the accuracy and efficiency of pharmaceutical research. Data quality, algorithmic bias, model interpretability, reproducibility, clinical translation barriers, and regulatory readiness present significant obstacles to effective implementation. The future of AI in pharmaceutical sciences appears to be highly promising. Emerging paradigms, including FL, GNNs, quantum computing integration, and XAI, are anticipated to enhance personalized medicine, multiomics analysis, and high-throughput drug screening. These technologies provide significant improvements in speed, scalability, and precision, potentially leading to substantial reductions in the time and cost associated with the market introduction of new therapeutics.

To fully leverage the potential of AI, robust interdisciplinary collaboration is necessary. Effective integration of computational predictions with experimental validation necessitates collaboration among data scientists, bioinformaticians, medicinal chemists, molecular biologists, clinicians, and regulatory experts. The establishment of standardized frameworks, transparent model evaluation protocols, and ethically grounded practices is essential for fostering trust and ensuring the safe, equitable, and impactful deployment of AI in real-world drug development contexts. In summary, AI catalyzes advancing the next generation of pharmaceutical innovation. The successful transition from code to clinical application will rely on technological advancements and the collaborative efforts of the scientific community.

## Figures and Tables

**Table 1:** Comprehensive representation of metrics for evaluating ML models.

S. no.	Metric name	Formula	Values range	Ideal values	Used in	Description and interpretation
1	Accuracy	((TP+TN)/(TP+TN+FP+FN))	0 to 1	Close to 1	C	It measures the ratio of the number of correctly predicted instances. The value of accuracy close to 1 will reflect a good model, but it can be misleading with imbalanced data.
2	Precision	(TP/(TP+FP))	0 to 1	Close to 1	C	It measures how many predicted positive instances are correct. A value of precision close to 1 will be good, and it also helps in minimizing false positives.
3	Error rate	1 − Accuracy	0 to 1	Close to 0	C	It defines the fraction of incorrect predictions. It is also a complement of accuracy.
4	Recall (sensitivity, TPR)	(TP)/(TP+FN)	0 to 1	Close to 1	C	It measures how many actual positives are correctly predicted. A robust ML model should have a recall (TPR) value near 1. It is good for minimizing false negatives.
5	Specificity (TNR)	(TN)/(TN+FP)	0 to 1	Close to 1	C	It measures how many actual negatives were correctly predicted. A robust ML model should have a TNR value near 1
6	Balanced accuracy	(TPR+TNR) /2	0 to 1	Close to 1	C	It is the average of recall and specificity. It is less biased for imbalanced classes.
7	F1-score	2 × (Precision × Recall) /(Precision+Recall)	0 to 1	Close to 1	C	It is a mean of precision and recall. The ideal value for a good ML model should be close to 1. It is a good balance for imbalanced data.
8	Matthews correlation coefficient (MCC)	$\left(\text{TP}\times \text{TN}-\text{FP}\times \text{FN}\right)/\sqrt{\left(\text{TP}+\text{FP}\right)\left(\text{TP}+\text{FN}\right)\left(\text{TN}+\text{FP}\right)\left(\text{TN}+\text{FN}\right)}$	−1 to 1	Close to 1	C	It is a balanced measure even for imbalanced datasets. An MCC value of 1: Perfect, 0: Random, and −1: Total disagreement.
9	Receiver operating curve–area under curve (ROC–AUC)	—	0.5 to 1	Close to 1	C	It is a plot among the true positive rate and the false positive rate. For a perfect ML model, it should be close to 1.
10	Precision–recall curve–area under the curve (PRC–AUC)	—	0 to 1	Close to 1	C	The computed PRC–AUC value should be close to 1 for a good ML model. It is more informative than ROC–AUC on imbalanced data
11	FPR (false positive rate)	(FP)/(FP+TN) OR 1 − specificity	0 to 1	Close to 0		It calculates the ratio of negative occurrences that are misclassified. It is a complement to specificity. If an FPR = 0 (no false positives), the model has perfect performance on the negative class. If an FPR = 1, it means all negative instances were misclassified as positive (bad model performance).
12	Cohen's kappa (*κ*)	((po − pe)/(1 − pe))	−1 to +1	Close to 1	C	It is an agreement score between predicted and true labels, adjusted for chance. If *κ* > 0.8, it indicates a strong agreement.
13	Confusion matrix	—	—	—	C	It tabulates TP, TN, FP, and FN counts for detailed insight. For a robust model, FN and FP should be as low as possible.
14	Mean absolute error (MAE)	1/N∑i=1Nyi−y^i	0 to ∞	Close to 0	Both C and R	It is an average of absolute errors. A perfect model will have an MAE close to 0.
15	Mean squared error (MSE)	1/N∑i=1Nyi−y^i2	0 to ∞	Close to 0	R	It is an average of squared errors. It penalizes large errors more than MAE and is sensitive to outliers. A perfect model will have an MSE close to 0.
16	Root mean squared error (RMSE)	$\sqrt{\text{MSE}}$	0 to ∞	Close to 0	R	It is the square root of MSE. A perfect model will have an RMSE close to 0.
17	Mean absolute percentage error (MAPE)	100/N×∑i=1Nyi−y^i/yi	0 to ∞	Close to 0	R	It is a percentage-based error metric. An ideal model should have MAPE close to 0.
18	*R* ^2^ score (coefficient of determination)	1−∑i=1Nyi−y^i2/∑i=1Nyi−y¯2	−∞ to 1	Close to 1	Both C and R	It is the proportion of variance explained by the model. A robust model will have an *R*^2^ close to 1. It can be negative if the model is worse than the mean prediction.
19	Adjusted *R*^2^ (*R*_adj_^2^)	1 − (1 − *R*^2^/*N* − *p* − 1)(*N* − 1)	−∞ to 1	Close to 1	R	*R*_adj_^2^ for the number of predictors and sample size. It is useful in feature selection.
20	Explained variance score	1−Varyi−y^/Varyi	−∞ to 1	Close to 1	R	Similar to *R*^2^, but does not penalize overfitting as strongly.
21	Applicability domain (e.g., PCA)	—	Inside/outside box prediction box	Prediction within the box	Both C and R	It ensures that a model only makes predictions for data similar to the training set. For a reliable ML model, the prediction should be inside the box. Outside-the-box predictions will be unreliable

*Note:* Actual value (*yi*), predicted value (y^i), mean of actual values ($\overset{¯}{y}$), number of samples (*N*), number of predictors (*p*), observed accuracy (*po*), expected agreement by chance (*pe*), classification model (C), regression model (R).

Abbreviations: FN, false negative; FP, false positive; TN, true negative; TP, true positive.

**Table 2:** Available tools for training and building the models.

Tool name	Year of development	Company/organization	Free/commercial	Python supported	GUI support	Website link	Used in biological work
Weka	2001	University of Waikato	Free	Yes (via Python wrapper)	Yes	https://www.cs.waikato.ac.nz/ml/weka/	Yes
Torch	2003	Ronan Collobert, Samy Bengio, Johnny Mariéthoz	Free	No	No	http://torch.ch/	No
Orange	2003	University of Ljubljana	Free	Yes	Yes	https://orange.biolab.si/	Yes/no
KNIME	2004	KNIME AG	Free	Yes	Yes	https://www.knime.com/	Yes
RapidMiner	2006	RapidMiner, Inc.	Free	Yes	Yes	https://rapidminer.com/	Yes
scikit-learn	2007	Community-Driven	Free	Yes	No	https://scikit-learn.org/	Yes
Infer.NET	2008	Microsoft Research	Free	Yes	No	https://dotnet.github.io/infer/	Yes/no
Apache Mahout	2008	Apache Software Foundation	Free	Yes	No	https://mahout.apache.org/	Yes
Theano	2010	Université de Montréal	Free	Yes	No	http://deeplearning.net/software/theano/	Yes
H2O.ai	2011	H2O.ai	Free	Yes	Yes	https://www.h2o.ai/	Yes
BigML	2011	BigML, Inc.	Commercial	Yes	Yes	https://bigml.com/	Yes
DataRobot	2012	DataRobot, Inc.	Commercial	Yes	Yes	https://www.datarobot.com/	Yes/no
Deeplearning4j	2013	Skymind	Free	Yes (via Python wrapper)	No	https://deeplearning4j.org/	Yes/no
Caffe	2013	Berkeley AI Research (BAIR)	Free	Yes (via Python wrapper)	No	http://caffe.berkeleyvision.org/	Yes
TensorFlow	2015	Google Brain Team	Free	Yes	No	https://www.tensorflow.org/	Yes
Keras	2015	Community-Driven	Free	Yes	No	https://keras.io/	Yes
MXNet	2015	Apache Software Foundation	Free	Yes	No	https://mxnet.apache.org/	No
Chainer	2015	Preferred Networks	Free	Yes	No	https://chainer.org/	No
PyTorch	2016	Facebook AI Research	Free	Yes	No	https://pytorch.org/	Yes
CNTK (Microsoft Cognitive Toolkit)	2016	Microsoft	Free	Yes	No	https://docs.microsoft.com/en-us/cognitive-toolkit/	Yes/no
Amazon SageMaker	2017	Amazon Web Services	Commercial	Yes	Yes	https://aws.amazon.com/sagemaker/	Yes
Teachable Machine	2017	Google	Free	No	Yes	https://teachablemachine.withgoogle.com/	Yes
IBM Watson Studio	2017	IBM	Commercial	Yes	Yes	https://www.ibm.com/cloud/watson-studio	Yes
ML.NET	2018	Microsoft	Free	Yes	No	https://dotnet.microsoft.com/apps/machinelearning-ai/ml-dotnet	No
Vertex AI	2021	Google Cloud	Commercial	Yes	Yes	https://cloud.google.com/vertex-ai/docs/training-overview	Yes/no

## Data Availability

No new data were generated for this review. All data discussed are derived from previously published studies, which are appropriately cited within the manuscript.
